# Beer and Consumer Response Using Biometrics: Associations Assessment of Beer Compounds and Elicited Emotions

**DOI:** 10.3390/foods9060821

**Published:** 2020-06-22

**Authors:** Claudia Gonzalez Viejo, Raúl Villarreal-Lara, Damir D. Torrico, Yaressi G. Rodríguez-Velazco, Zamantha Escobedo-Avellaneda, Perla A. Ramos-Parra, Ronit Mandal, Anubhav Pratap Singh, Carmen Hernández-Brenes, Sigfredo Fuentes

**Affiliations:** 1 Digital Agriculture, Food and Wine Sciences Group, School of Agriculture and Food, Faculty of Veterinary and Agricultural Sciences, University of Melbourne, Melbourne, VIC 3010, Australia; sfuentes@unimelb.edu.au; 2 Tecnologico de Monterrey, Escuela de Ingeniería y Ciencias, Ave. Eugenio Garza Sada 2501, Monterrey 64849, N.L., Mexico; raulvl@tec.mx (R.V.-L.); A00801965@itesm.mx (Y.G.R.-V.); zamantha.avellaneda@tec.mx (Z.E.-A.); perlaramos@tec.mx (P.A.R.-P.); chbrenes@itesm.mx (C.H.-B.); 3 SensoLab Solutions, Centro de Innovación y Transferencia Tecnológica (CIT2), Ave. Eugenio Garza Sada #427 Col. Altavista, Monterrey 64849, N.L., Mexico; 4 Department of Wine, Food and Molecular Biosciences, Faculty of Agriculture and Life Sciences, Lincoln University, Lincoln 7647, New Zealand; damir.torrico@lincoln.ac.nz; 5 Food, Nutrition, and Health, Faculty of Land and Food Systems, University of British Columbia, 2205, East Mall, Vancouver, BC V6T 1W4, Canada; ronit123@mail.ubc.ca (R.M.); anubhav.singh@ubc.ca (A.P.S.)

**Keywords:** hordenine, happiness, beer consumption, sensory analysis, beer styles

## Abstract

Some chemical compounds, especially alcohol, sugars, and alkaloids such as hordenine, have been reported as elicitors of different emotional responses. This preliminary study was based on six commercial beers selected according to their fermentation type, with two beers of each type (spontaneous, bottom, and top). Chemometry and sensory analysis were performed for all samples to determine relationships and patterns between chemical composition and emotional responses from consumers. The results showed that sweeter samples were associated with higher perceived liking by consumers and positive emotions, which corresponded to spontaneous fermentation beers. There was high correlation (*R* = 0.91; *R^2^* = 0.83) between hordenine and alcohol content. Beers presenting higher concentrations of both, and higher bitterness, were related to negative emotions. Further studies should be conducted, giving more time for emotional response analysis between beer samples, and comparing alcoholic and non-alcoholic beers with similar styles, to separate the effects of alcohol and hordenine. This preliminary study was a first attempt to associate beer compounds with the emotional responses of consumers using non-invasive biometrics.

## 1. Introduction

Beer is a complex alcoholic beverage in terms of its chemical composition and ingredients, such as barley, yeast, hops, and, in some beer products, includes adjuncts that may consist of other cereals or fruits [1,2]. The wide range of combinations that may be used from each of the ingredients, along with the differences in brewing methods, have a great influence on the development of beer’s chemical and aroma profiles. Among the most important beer quality compounds are the iso-alpha acids from hops, which are responsible for the bitterness characteristic of the final product, proteins from yeast and barley, which contribute to the foamability and foam stability, and alcohol/sugar content, which determine the strength of beer [3,4,5]. Furthermore, beer also contains inorganic salts, alkaloids, polyphenols, aminoacids, and hop resins, which affect the physical and sensory characteristics of the final product [6]. Beer is a beverage product that potentially affects human emotional responses elicited by its chemical components.

Some chemical compounds in foods have been associated with either the suppression or release of certain neurotransmitters that trigger different emotions in humans. Most of these components are alkaloids, which are mainly considered as biological amines, but may present a diversity of structures in the form of esters or amides, or combined with sugars [7,8]. Cacao contains alkaloids such as theobromine, caffeine, phenylethylamine, and salsolinol, which have been studied for their psychoactive effects on humans. The synergistic effect of theobromine and caffeine has been associated with changes in mood such as energetic arousal and an increase in cognitive function, as well as changes in physiological responses such as heart rate [9,10]. Phenylethylamine is a compound similar to amphetamines, which triggers serotonin that regulates the mood and has been associated with emotions such as joy, happiness, and love [11,12]. Furthermore, salsolinol is able to bind with dopamine receptors and stimulate the release of endorphins, which leads to a sensation of reward and suppression of pain [9]. On the other hand, other chemical compounds, such as alcohol, cause a reduction in serotonin levels, which leads to depression [13].

Beer also contains some alkaloids, such as salsolinol and hordenine, the latter being in higher concentrations than salsolinol [14,15]. Hordenine is naturally found in barley during its germination; therefore, it is passed through the beer process in malting [16]. It has been reported that the hordenine concentration in beer mainly depends on the time, temperature, and humidity during the germination process of barley [17]. Even though it is found in low to moderate concentrations in beers, this alkaloid contributes to the diuretic effect of beer and provides some bitterness characteristics [16,18]. Regarding the effects of hordenine in humans, it has been reported to increase heart rate and blood pressure [19] as well as to stimulate the release of dopamine, which has been related to happiness [15,20]. On the other hand, other authors have concluded that beer flavors are the main factor responsible for dopamine release [21]. However, first study [15] was conducted by testing hordenine in radioligand assays, but not testing the compound in beer, while the second study [21] consisted in performing a positron emission tomography (PET) when tasting beer flavors. Hence, those experiments did not evaluate the effects of beer on consumers’ emotional responses. Thus, there is still a large gap in the understanding of the mechanisms behind beer tasting and consumer perceptions.

This study aimed to assess the effect of beer compounds on the emotional responses of consumers using traditional sensory tests (self-reported responses) as well as non-invasive biometrics (unconscious responses). For these purposes, six beer samples from different fermentation types were used to measure the physicochemical data such as color, iso-alpha acids, hordenine, alcohol content, and bitterness, among others. Furthermore, a sensory session was conducted with Mexican beer consumers to obtain both self-reported and subconscious responses in order to assess their acceptability and elicited emotions. Multivariate data analysis was conducted to assess the relationship between the physicochemical, liking, and emotional responses from consumers.

## 2. Materials and Methods

Six commercial beer samples, two from each of the three different types of fermentation (Table 1), were selected from a pool of 24 beers previously analyzed for physicochemical and sensory descriptors [1,3,22,23,24,25]. The number of samples was limited to six as this is the recommended maximum number to avoid consumers’ fatigue, especially due to alcohol content and bitterness, and this may also lead to a decrease in the quality of the responses [26,27]. Samples were purchased from a local supplier (Beer for Us S.A. de C.V., Monterrey, NL, México) and stored as described. For physicochemical characterization, samples were divided into aliquots and stored at −80 °C until their use, while samples for sensory evaluations were kept first at room temperature and then put in refrigeration (4 °C) 24 h before tests were conducted. Samples were defrosted at 4 °C and, prior to physicochemical analysis, were degassed using an ultrasound bath (SH30H, Elmasonic, Frechen, Germany) at 37 kHz for 30 min.

### 2.1. Physicochemical Characterization

The color parameters (L*, a*, b*) were measured using a LabScan XE System (Hunter Associates Laboratory, Inc., Reston, VA, USA) colorimeter by triplicate using a CIELAB system. Instrumental color was expressed as the Hue angle (Equation (1)), Chroma (Equation (2)), and Yellowness Index (YI, Equation (3); [28]). Evaluation of viscosity was determined in triplicates using a rheometer (MCR 302, Anton Paar Canada Inc, Quebec, Canada) at 25 °C. All samples (30–40 mL) were measured for pH at 25 °C in triplicates using a Fisherbrand Accumet^®^ AB15 pH meter (Fischer Scientific, Hampton, NH, USA) calibrated against standard buffers (3-point calibration: pH 4.00–0.1 M Potassium hydrogen phthalate buffer, pH 7.00—Potassium Phosphate Monobasic/Sodium Hydroxide buffer, pH 10.00–Potassium Carbonate/Potassium Tetraborate/Potassium Hydroxide/Disodium EDTA Dihydrate buffer). Titratable acidity (TA, as % acetic acid) was measured in duplicates, with the method described by Okafor et al. [29], and the following modifications. A total of 5 mL was titrated with 0.1 M NaOH until pH = 7.0 was achieved using bromothymol blue as an indicator.

(1) $$\mathrm{Hue}\;\mathrm{angle}=\arctan(\frac{b*}{a*})\times \frac{180}{3.14}$$

(2) $$\mathrm{Chroma}={\left({a*}^{2}+{b*}^{2}\right)}^{0.5}$$

(3) $$\mathrm{YI}=142.86\;\times \frac{b*}{L*}$$

The density of samples was assessed based on weight and volume (50 mL). Total dissolved solids (TDS) were measured in triplicates using a Yuelong YL-TDS2-A digital water quality tester (Zhengzhou Yuelong Electronic Technology Co., Ltd., Zhengzhou City, Henan Province, China). Salt concentration was obtained using two drops of the sample in triplicates added to a digital salt-meter (PAL-SALT Mohr, Atago Co., Ltd. Saitama, Japan). On the other hand, alcohol content was assessed using 18 mL of the sample at room temperature (20 °C) injected to an Alcolyzer Wine M alcohol meter (Anton Paar GmbH, GRAZ, Austria) with the wine extension method found in the equipment settings; the instrument has a maximum error of 0.1% vv^−1^.

### 2.2. Characterization of Simple Sugars by HPLC-Refractive Index

The simple sugars profile was measured as described by Heredia-Olea et al. [30] and Alonso-Gómez et al. [31] with slight modifications. The samples were filtered through a polyvinylidene fluoride (PVDF) syringe filter (0.2 µm) and injected into high-performance liquid chromatography (HPLC) equipment (Waters HPLC Breeze model, Waters, Milford, MA, USA) with a refractive index detector (Waters 2414) kept at 50 °C. The chromatographic separation was achieved using an ion-exclusion column Phenomenex Rezex ROA-organic acid h+ (250 × 4.6 mm, 8 µm particle size, Phenomenex, Torrance, CA, USA) at 60 °C. The mobile phase consisted of a 5 mM H_2_SO_4_ solution with a 20 min isocratic flow rate of 0.4 mL min^−1^ and with an injection volume of 10 μL. Glucose, maltose, and fructose quantifications were performed with calibration curves of HPLC-grade standards (Sigma-Aldrich, St. Louis, MO, USA).

Calibration curves for glucose (1–25 mg mL^−1^), maltose (0.5–5 mg mL^−1^), and fructose (1–25 mg mL^−1^) of HPLC-grade standards (Sigma-Aldrich, St. Louis, MO, USA) were constructed for quantification purposes (data not shown).

### 2.3. Determination of Bitterness

Bitterness was assessed by manual isooctane extraction as described in the American Society of Brewing Chemists (ASBC) Methods of Analysis with the following modifications [32]. A total of 5 mL of beer was acidified with hydrochloric acid (HCl; 0.5 mL, 3M) and isooctane (10 mL); subsequently, it was homogenized for 15 min using a mechanical shaker. The separation of organic and aqueous layers was performed by centrifugation at 400 *g* × 5 min. Finally, the isooctane phase (upper) was measured spectrophotometrically at 275 nm. A calculation of bitterness units (IBU) of beer was obtained, as shown in Equation (4).

(4) $$\mathrm{IBU}=absorbance_{275}\times 50$$

### 2.4. Characterization of Iso-α-Acids

The iso-α-acids profile was analyzed as described by Vanhoenacker et al. [33]. The beer samples (~25 mL) were filtered through a polytetrafluoroethylene (PTFE) syringe filter (0.2 µm) and directly injected to an Acquity Ultra-Performance Liquid Chromatography (UPLC, Waters, Milford, MA, USA) coupled to Diode-Array Detector (DAD), monitoring at 270 nm. The chromatographic separation was achieved using a Zorbax Extend C-18 column (100 × 3 mm, 3.5 µm particle size, Agilent, Santa Clara, SA, USA) kept at 35 °C. The mobile phase consisted of 5 mM ammonium acetate in 20% ethanol (pH 9.95) as phase A; acetonitrile/ethanol (60:40 v/v) as phase B. The solvent flow rate was 0.4 mL min^−1^, using gradients of 0−3 min, 0% B; 3–4 min, 0−16% B; 4–54 min, 16–40% B; 54–57 min, 40–95% B; 57–65 min, 95%B; 65–67 min, 95–0% B, followed by 20 min re-equilibration. The iso-α-acids quantification was performed with calibration curves of commercial standards of trans-iso-α-acids in dicyclohexylamine (DCHA) obtained from the American Society of Brewing Chemists (Saint Paul, MN, USA). The standards were prepared in methanol (0.05% H_3_PO_4_) according to the supplier’s recommendations and the ASBC Methods of Analysis [34].

### 2.5. Hordenine Determination by UPLC-MS/MS

Hordenine sample preparation was performed as described by Sommer et al. [35] with slight modifications. Beer samples were centrifuged for 15 min at 12,000× *g* and 4 °C; two dilution steps were followed. *Dilution I (Dil. I):* 50 μL of degassed beer were added to 450 μL of 0.1% formic acid. *Dilution II (Dil. II)*: 20 μL of *Dil. I* were added to 980 μL of 0.1% formic acid. The solutions obtained after *Dil. II* were passed through a PVDF filter (0.2 μm, Thermo Scientific™, Waltham, MA, USA) prior to the analysis. For quantification, a calibration curve with a range of 0–0.1 ppm was developed using a stock solution (2 mg mL^−1^) of a hordenine commercial standard (Sigma-Aldrich, St. Louis, MO, USA) prepared in formic acid (0.1%).

Hordenine separation and quantification were conducted in a Quattro Premier XE Micromass UPLC-MS/MS system (Waters, Milford, MA, USA) equipped with a triple quadrupole mass spectrometer (QQQ-MS) connected to an Acquity UPLC (Waters, Milford, MA, USA) with electrospray ionization (ESI) source in positive mode. Hordenine was analyzed in the multiple reaction monitoring (MRM) mode of m/z 165.95:121. Masslynx 4.1 software (Waters, Milford, MA, USA.) was used for data acquisition and instrument control. Hordenine separation was performed using a high strength silica (HSS T3 C18) column (2.1 mm × 100 mm, 1.8 μm particle size) coupled with a VanGuard HSS T3 C18 column (2.1 × 5 mm, 1.8 μm) maintained at 50 °C. Mobile phases were 0.1% formic acid in water (solvent A) and 0.1% formic acid in 70% acetonitrile and 30% methanol (solvent B) with 6.6 min total gradient solution as follows: 0–1 min, 5–15% B; 1–2 min, 15–40% B; 2–3 min, 40–70% B; 3–3.5 min, 70–100% B; 3.5–5 min, 100% B; 5–5.2 min, 100–5% B, followed by 1.4 min re-equilibration. The flow rate was kept constant at 0.5 mL min^−1^ with an injection volume of 10 μL. Nitrogen was used as the desolvation gas (400 L/h). The selected ion monitoring conditions were set as capillary voltage 2.5 kV, source temperature 120 °C, and desolvation temperature 400 °C. All determinations were conducted in triplicates.

### 2.6. Consumer Sensory Evaluation and Biometrics

A sensory session was carried out in Monterrey, NL, Mexico, which is the state with the highest alcoholic drinks consumption with beer as the leader [36,37]. The session was conducted with *N* = 61 beer consumers (frequency > three times a month; 54% males; 46% females) between 18 and 51 years old (mean age 25.6 ± 6.9 years). Participants were recruited via email and asked to participate in a graduate research project from the Department of Bioengineering, School on Engineering and Sciences of Tecnológico de Monterrey, Campus Monterrey, Mexico (Ethics ID: CSERDBT-0002). According to the Power analysis conducted using the Power and Sample Size Calculator from the SigmaXL ver. 8.15 software (SigmaXL Inc., Kitchener, ON, Canada), the number of participants was sufficient to find significant differences (1-β = 0.98) among the beer samples. The session was conducted at SensoLab Solutions SC, a sensory and consumer science laboratory center, located at the Technology Transfer and Innovation Center of Tecnológico de Monterrey, Mexico. The laboratory was equipped with eight individual sensory booths with uniform lighting. Each booth had an Android^®^ (Google, Mountain View, CA, USA) Samsung Galaxy Tab 4 tablet (Samsung, Seoul, South Korea) displaying the Bio-Sensory application (App; The University of Melbourne, Parkville, Vic, Australia). The App was able to present the questionnaire (Table 2) and record videos from the participants while tasting the beer samples to further analyze their emotional responses [29]. Samples (30 mL) were served at refrigeration temperature (4 °C), and water was used as palate cleanser before and between each sample. To assess the visual descriptors of the beers, a video showing the pouring of the sample using the RoboBEER (The University of Melbourne, Parkville, Vic, Australia) was displayed in the App to avoid bias from the variability due to the pouring method and glass effects [22]. As shown in Table 2, two overall liking ratings were obtained at the start and end of the tasting to verify if there is a bias on this descriptor based on the evaluation of specific attributes.

Videos were analyzed using an application developed based on the Affectiva software development kit (SDK; Affectiva, Boston, MA, USA). This application uses the histogram of the oriented gradient to detect and track the micro- and macro-movements of face features and is able to evaluate all videos in batch. Furthermore, it is capable of assessing facial expressions using support vector machine algorithms to translate them into emotions such as (i) contempt, (ii) disgust, (iii) sadness, (iv) surprise, (v) joy, (vi) valence, (vii) engagement, and (viii) attention, as well as emojis related to facial expressions such as (ix) smiley 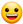, (x) relaxed 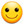, (xi) winking face 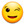, (xii) stuck out tongue 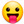, (xiii) flushed 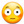, (xiv) rage 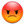, (xv) smirk 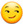, and (xvi) disappointed 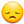 [39].

### 2.7. Statistical Analysis

All data were analyzed through ANOVA and least significant differences (LSD) as a post-hoc test (α = 0.05) using Minitab 17.2.1 (Minitab Inc., State College, PA, USA). A linear correlation analysis was conducted for alcohol and hordenine values using Microsoft Excel (Microsoft, Redmond, WA, USA). Chemical, sensory (self-reported), and biometric responses were assessed using multivariate data analysis based on principal components analysis (PCA), and multiple factor analysis (MFA) with a customized code written in Matlab^®^ R2019b (Mathworks, Inc., Natick, MA, USA) and XLSTAT ver. 2020.1.1 (Addinsoft Inc., New York, NY, USA), respectively.

## 3. Results

### 3.1. Physicochemical Results

Table 3 shows the mean values and results from the ANOVA for selected physicochemical parameters. There were significant differences (*p* < 0.05) between samples for all parameters. Sample Z had the lowest mean value for L* (26.58) as this is the darkest beer, while C had the highest value (59.36). Similarly, the yellow index (YI) was higher for Z (200.40) than all other samples, C being the lowest (15.69). Spontaneous fermentation beers (LK and LF) were the highest in density (1.02 and 1.03 g mL^−1^, respectively), and significantly different from the other samples. On the other hand, LK was the most viscous (2.16 mPa s), followed by Z and H (1.80 mPa s), with C as the least viscous (1.48 mPa s). On the other hand, the spontaneous fermentation samples were the most acidic (LF: pH = 2.94, TA = 0.32; LK: pH = 3.17, TA = 0.41), while Z was the least acidic (pH = 4.42, TA = 0.17).

**Figure 1:**
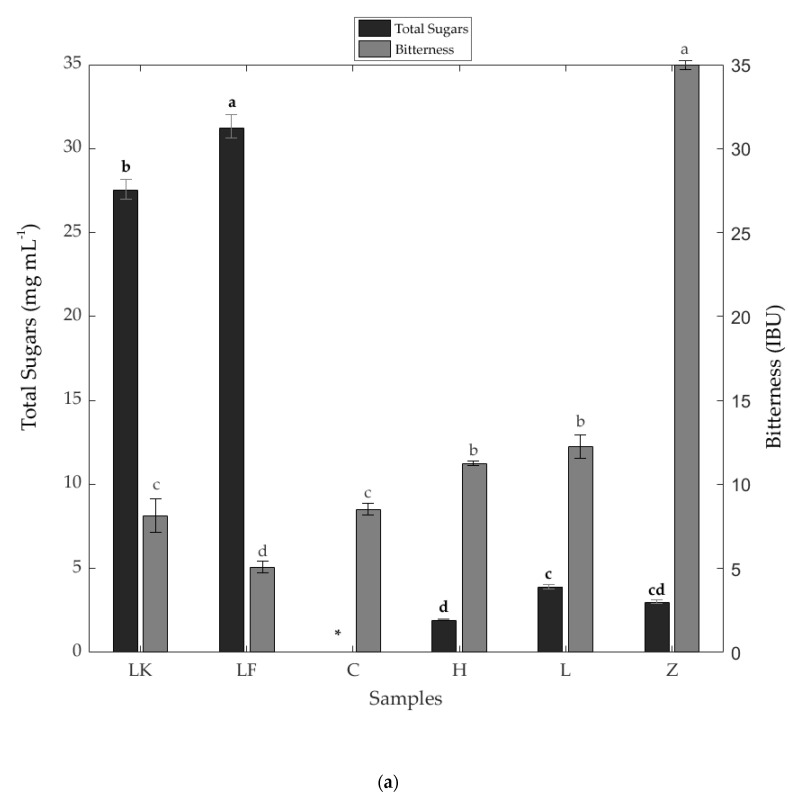

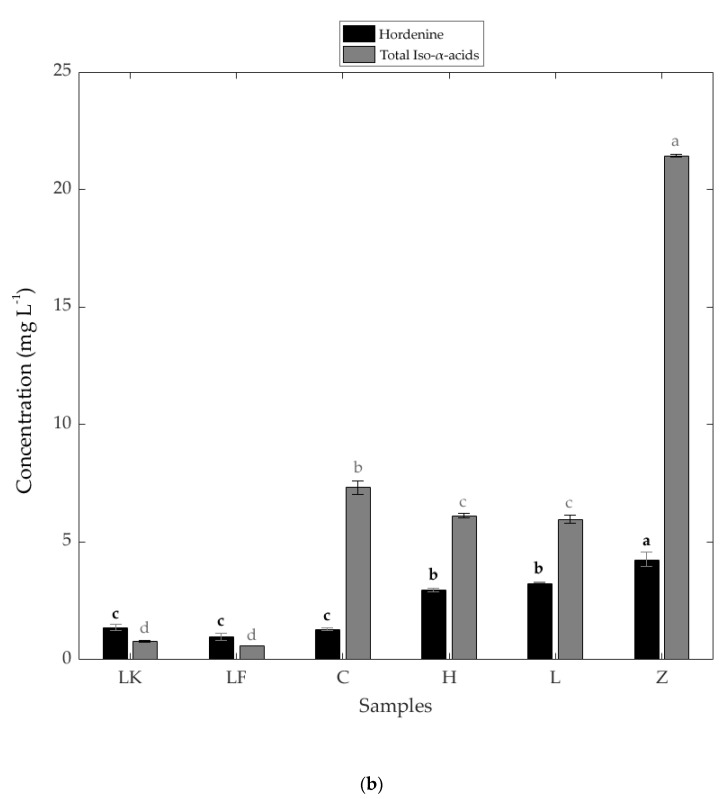
Chemical characterization of commercial beers, including (**a**) total sugars (mg mL^−1^), bitterness (IBU), (**b**) hordenine (mg L^−1^), total Iso-α-acid concentration (mg L^−1^). Different letters above bars denote significant differences between beer samples, for the same chemical parameter, according to the least significant difference test (LSD; *p* < 0.05). * Total sugars not detected in beer C. All values are the mean ± SE (error bars) of independent determinations. *n* = 3, hordenine, and bitterness; *n* = 2, total sugars, and total-α-acids. Abbreviations of samples may be found in Table 1.

Figure 1 shows the means and ANOVA results of the total sugars, bitterness (Figure 1a), iso-alpha acids, and hordenine (Figure 1b). The spontaneous fermentation beers had significantly higher (*p* < 0.05) total sugar content (LF: 31.23 mg mL^−1^; LK: 27.53 mg mL^−1^) than the samples from other types of fermentation; for C, the sugar concentration was non-detectable with the chromatographic conditions used. Sample Z was the highest in both bitterness (34.98 IBU) and total iso alpha-acids (21.41 mg L^−1^), while LF was the least bitter (bitterness: 5.08 IBU; total iso-alpha acids: 0.60 mg L^−1^). On the other hand, the top fermentation beers (Z and L) had the highest concentrations of hordenine (Z: 4.24 mg L^−1^; L: 3.22 mg L^−1^), while spontaneous fermentation sample LF had the lowest content (0.98 mg L^−1^).

Table 4 shows that the simple sugars from the spontaneous fermentation samples (LF and LK) were mainly composed of glucose (LF: 14.32 mg mL^−1^; LK: 13.91 mg mL^−1^), followed by fructose (LF: 13.51 mg mL^−1^; LK: 12.56 mg mL^−1^), and maltose (LF: 3.40 mg mL^−1^; LK: 1.06 mg mL^−1^). Sample H had higher values of maltose (0.79 mg mL^−1^) than glucose (0.60 mg mL^−1^) and fructose (0.50 mg mL^−1^), while L was higher in fructose (2.04 mg mL^−1^) than glucose (1.87 mg mL^−1^) and did not contain maltose. Spontaneous fermentation beers were the highest in salt concentration (LK and LF: 0.10%), while C was the lowest (0.05%). A similar trend was found for TDS with LF and LK; although being significantly different, both presented the highest values (LF: 1226 ppm; LK: 1148 ppm), while C had the lowest with 658 ppm. Top fermentation beers showed the highest alcohol content (Z: 9.47%; L: 6.68%), while spontaneous fermentation samples had the lowest (LF: 2.53%; LK: 3.53%). A similar trend was found for the content of trans-Isocohumulone and trans-Isohumulone parameters with Z being the highest concentration (10.95 mg L^−1^, and 10.46 mg L^−1^, respectively), and LF the lowest (0.22 mg L^−1^, and 0.38 mg L^−1^, respectively).

### 3.2. Consumer Sensory Evaluation and Biometrics

Table 5 shows the mean values and ANOVA results of the self-reported responses from the consumers’ sensory tests. Significant differences (*p* < 0.05) between samples were observed for all attributes evaluated. In all samples, except for Z, the responses from overall liking were higher when rated at the end of the test after assessing each attribute, compared to the overall liking at the start (before assessing individual attributes). Spontaneous fermentation beers with raspberry (Framboise) and cherry (Kriek) flavors were the most liked overall (LF: 10.79; LK: 10.73) and also received the highest in bitterness (LF: 11.85; LK: 11.06), acidity (LF: 10.76; LK: 11.37) and aroma (LF: 9.50; LK: 9.53) liking scores. For sweetness liking, there were non-significant differences among the spontaneous (LK, LF) and bottom fermentation samples (C, H), but these were significantly different from the top fermentation beers (L, Z). On the other hand, C had the lowest liking of foam stability (6.79) compared to all other beers (10.20–11.14).

**Figure 2:**
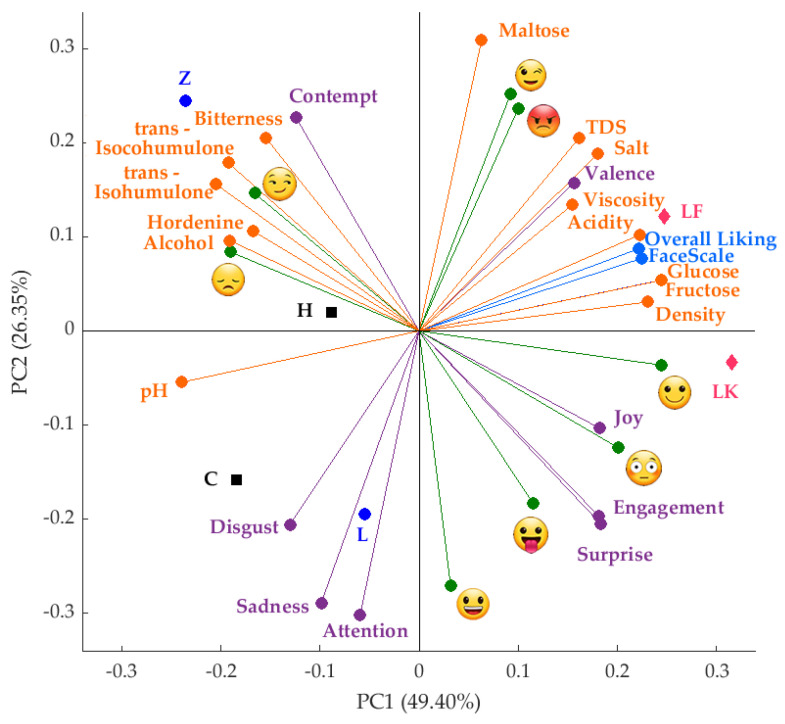
Principal components analysis showing the results from the sensory (self-reported liking responses in blue), biometrics (purple for emotions, and green for the emojis generated from the facial expressions), and chemical data (orange). Abbreviations: PC1 and PC2 = principal component 1 and 2, TDS = total dissolved solids. Abbreviations for samples are shown in Table 1.

Figure 2 shows the principal components analysis for the sensory self-reported and emotional (biometric) responses, and chemical data. The principal component one (PC1) represented 49.40%, while PC2 accounted for 26.35% of data variability (Total = 75.75%). According to the factor loadings (FL), descriptors such as relaxed 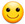 (FL = 0.24), glucose (FL = 0.24), fructose (FL = 0.24) and density (FL = 0.23) represented PC1 on the positive side of the axis; while pH (FL = −0.24), trans-Isohumulone (FL = −0.20) and trans-Isocohumulone (FL = −0.19) characterized it on the negative side. On the other hand, PC2 was represented by maltose (FL = 0.31), winking face 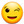 (FL = 0.25) and rage 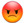 (FL = 0.24) on the positive side; while attention (FL = −0.30), sadness (FL = −0.29), and smiley 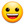 (FL = −0.27) represented it on the negative side of the axis. Sugars such as fructose and glucose were positively related to overall liking, FaceScale and relaxed, with the spontaneous fermentation beers (LK and LF) associated with those components. On the contrary, hordenine presented a negative relationship with the latter descriptors and a positive relationship with alcohol content, iso-alpha acids, bitterness, smirk 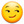, and disappointed 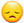, and beers such as H (bottom fermentation) and Z (top fermentation) were associated with these variables.

**Figure 3:**
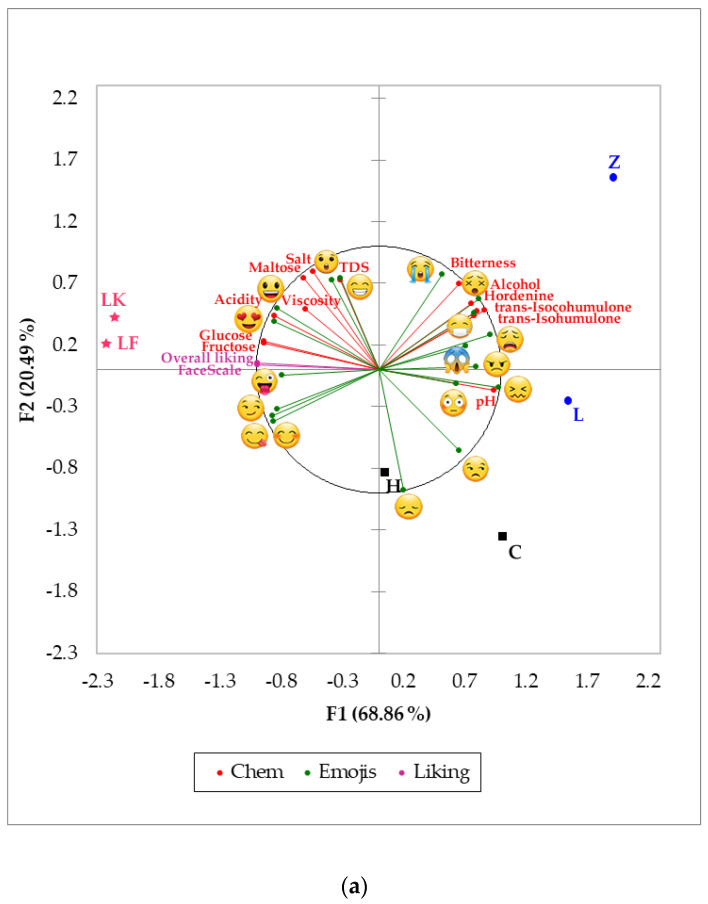

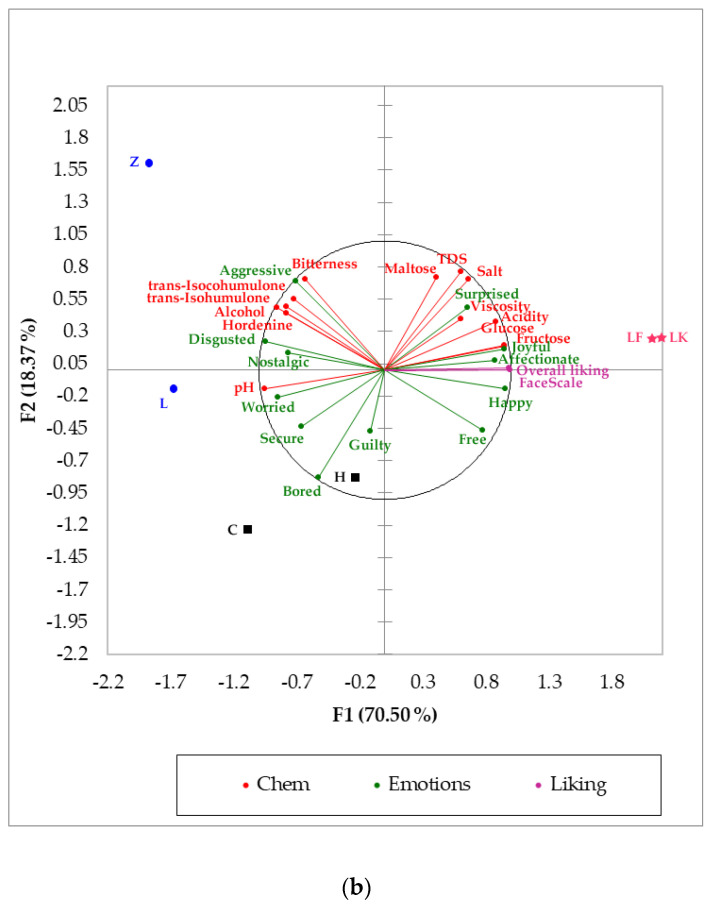
Multiple factor analysis showing the results from the sensory (self-reported liking responses), chemical data (Chem), and (**a**) emoji and (**b**) emotions, check all that apply responses. Abbreviations: F1 and F2 = Factor 1 and 2, TDS = total dissolved solids. Abbreviations for samples are shown in Table 1.

Figure 3 shows the MFA for all chemicals, liking, and check all that apply data using emojis (Figure 3a) and emotion-terms (Figure 3b). In the MFA using emojis (Figure 3a), it can be observed that factors 1 and 2 (F1 and F2) represented 89.35% of total data variability (F1 = 68.86%; F2 = 20.49%). According to FL, the F1 was mainly represented by crying 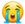 (FL = 1.13), pH (FL = 0.94), angry 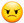 (FL = 0.88), and alcohol content (FL = 0.87) on the positive side of the axis, and by overall liking (FL = −0.99), FaceScale (FL = −0.99), glucose (FL = −0.94) and fructose (FL = −0.94) on the negative side. On the other hand, F2 was represented by crying 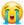 (FL = 1.67), TDS (FL = 0.80), salt (FL = 0.74) and maltose (FL = 0.73) on the positive side, and by disappointed 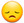 (FL = −0.50) and unamused 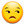 (FL = −0.29) on the negative side of the axis. Hordenine was positively related to alcohol content, iso-alpha acids, bitterness, and emojis such as sick 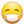, dizzy 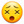 and weary 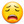, with top fermentation beer samples such as Z and L associated with those variables. In contrast, overall liking and FaceScale had a positive relationship with glucose, fructose, winking face with tongue 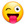, and love 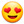; spontaneous fermentation samples LK and LF were most represented by these descriptors.

In Figure 3b, developed using emotion-terms, F1 and F2 accounted for 88.87% of total data variability (F1 = 70.50%; F2 = 18.37%). Based on the FL, variables such as FaceScale (FL = 0.99), overall liking (FL = 0.99), glucose (FL = 0.95) and fructose (FL = 0.95) represented the positive side of the F1 axis, while pH (FL = −0.95), acidity (FL = −0.88), aggressive (FL = −0.86), and alcohol content (FL = −0.86) characterized the negative side. Descriptors such as aggressive (FL = 0.84), TDS (FL = 0.77), maltose (FL = 0.72) and bitterness (FL = 0.71) represented the positive side of the F2 axis, whereas bored (FL = −0.54) and guilty (FL = −0.20) characterized the negative side. Similar to Figure 3a, hordenine had a positive relationship with alcohol content, iso-alpha acids, bitterness and emotion-terms such as aggressive, disgusted and nostalgic. These were negatively related with overall liking, FaceScale, fructose, glucose, acidity, joyful, affectionate, and happy. Samples were clearly grouped according to the type of fermentation: top (Z and L), bottom (C and H) and spontaneous (LF and LK).

## 4. Discussion

Spontaneous fermentation beers resulted in the highest values for total sugars and lowest alcohol content, bitterness (expressed as IBU), and iso-alpha acids (Figure 1). This may be due to the addition of fruit juice (cherry in LK and raspberry in LF), and dried hops, which may also be old and oxidized to provide aromas and flavors but not bitterness [23,25,40].

There was a positive correlation (*R* = 0.91; *R^2^* = 0.83) between hordenine and alcohol content for all beer samples studied. The latter effect is in accordance with the study from Brauers et al. [16], who found higher hordenine content in strong beers (bock style), which have high alcohol content (6.6–7.5%; [41]), and lower hordenine values in alcohol-free beers. On the other hand, top fermentation beers were found to have higher concentrations of iso-alpha acids, hordenine, and bitterness (expressed as IBU) compared to the other samples (Figure 3). Sensorial bitterness can be derived from several compounds, including polyphenols and alkaloids [42].

For beers, 80% of the perceived bitterness is originated from adding hops during the brewing process [43]. Hops from female plants contain glands with a resin that is rich in derivates of phloroglucinol, essential oils, and flavonoids [44]. In terms of the bitter compounds, there are two types of acids in the hops resins, alpha, and beta; however, these molecules are not bitter in their raw forms. Before brewing, a thermal isomerization of the alpha-acids occurs during the boiling process, and iso-alpha acids are obtained, which are responsible for imparting the bitterness in beer. Two stereoisomers are generated during this isomerization process, trans- and cis-iso-alpha-acids, which are catalyzed by magnesium ions [45]. The perceived bitterness intensity is higher when there is a higher content of iso-alpha-acids. This compound provides a “harsh,” “round,” and “lingering” flavor to beer [43]. In the present study, the top fermentation beers (L and Z) had the lowest scores for the liking of bitterness compared to the other beer samples (Table 5). The higher chemical bitterness (expressed as IBU) for these two samples can potentially explain the disliking of the bitterness in the tasting session by the participants. Besides, hordenine is known to impart bitterness [18], and the concentration of this compound was also higher in the top fermentation beers.

Similar results were found using the conscious responses with emojis and words, and from the subconscious responses using biometrics. According to the PCA and MFA presented in Figure 2 and Figure 3, respectively, beers with higher sugar content (glucose and fructose) were associated with positive emotions such as joy, relaxed 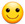, love 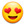, winking face with tongue 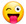, affectionate, and FaceScale in both subconscious and conscious responses (emojis and emotion-terms). This coincides with findings by Kim et al. [46], who reported that samples of beverages and biscuits with the highest sugar content elicited positive emotions such as affectionate, pleased, joyful, glad, and happy. On the other hand, bitterness has been associated with rejection due to genetic factors and the innate relationship of bitter products with poisonous compounds [24,47,48]. Overall taste liking is the result of the intrinsic balance among the basic tastes that are sensed by the receptors located in the gustative system [49]. Individual taste compounds can elicit discrete sensations in consumers. However, different tastes can interact with each other, which can result in suppression or enhancement effects of certain perceptions [50,51]. For instance, minor concentrations of sugar can enhance the sourness of citric acid solutions; or slight concentrations of salt can enhance the sweetness of sugar solutions. The opposite can also occur as slight concentrations of quinine (a bitter compound) mixed with saccharides can suppress the sweetness of the solutions [52,53]. This can potentially explain the overall taste perception by the consumers in the present study. As the sugar content of the spontaneous fermentation beers was higher compared to the other samples, the bitterness perception of those beers was somewhat suppressed, which produced higher hedonic and emotional responses. This effect can be observed for both responses (conscious and subconscious) measured in this study, as the sweet taste was the main factor responsible for the overall satisfaction of consumers.

Even though hordenine has been reported to stimulate the release of dopamine and is, therefore, associated with happiness [15,21], these studies have not evaluated these effects on consumers when drinking beer. In the present research, it was found that, as hordenine was positively related with bitterness and other bitter compounds such as iso-alpha acids, all these had a positive relationship with negative emotions such as disappointed 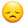 (Figure 2), dizzy 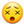, sick 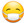, weary 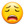
(Figure 3a), disgusted, and aggressive (Figure 3b). This may be due to two main factors: i) the higher sugar concentration in beers LF and LK, which had a higher effect on consumers, and ii) the time of the sensory session, which may not have been long enough to increase hordenine concentration in the bloodstream significantly. Hence, since the effects of hordenine may be delayed, a sensory tasting session, including several sample beers, may not be appropriated to study the carry-over effects. This may be overcome by conducting further research allowing more time between beers for emotional assessments, so that there is enough hordenine level in the blood to more accurately assess the elicited emotional responses. Moreover, by comparing similar beer styles with alcoholic and non-alcoholic beers, it may render more information on the effects of hordenine and other compounds alone.

## 5. Conclusions

This preliminary study was a first attempt to associate beer compounds with the emotional responses of consumers using non-invasive biometrics. Findings showed that there was a positive relationship between sugar content, acidity, and positive emotions. At the same time, alcohol, bitterness, and hordenine were associated with negative emotions, which explain the consumers’ preference for spontaneous fermentation samples, which are sweeter and less bitter than other beer styles. The strong correlation between alcohol and hordenine, along with the effect that time may have in terms of increasing the hordenine levels in the bloodstream, leads to the need to conduct further studies, which may allow giving more time between samples to assess emotional responses and to compare alcoholic and non-alcoholic beers with similar styles to separate the effects of alcohol and hordenine. Additionally, further studies may include the assessment of differences in emotional responses among consumers from different cultural backgrounds. Results from these studies may be useful for brewing companies to modify their products for different markets and satisfy the needs of distinct target consumers.

## Figures and Tables

**Table 1:** Beer styles and labels of samples used to report results.

Beer Style	Beer Fermentation	Country of Origin	Label
Lambic Kriek	Spontaneous	Belgium	LK
Lambic Framboise	Spontaneous	Belgium	LF
Pale Lager	Bottom	Mexico	C
Pale Lager	Bottom	Mexico	H
Blonde Ale	Top	Belgium	L
Porter	Top	Poland	Z

**Table 2:** Questionnaire presented in the Bio-Sensory application.

Question/Descriptor	Answers (Options)	Scale
Overall liking (rated at the start of the test)	Dislike extremely—Like extremely	15-cm non-structured scale
Foam stability	Dislike extremely—Like extremely	15-cm non-structured scale
Foam height	Dislike extremely—Like extremely	15-cm non-structured scale
Bitterness	Dislike extremely—Like extremely	15-cm non-structured scale
Sweetness	Dislike extremely—Like extremely	15-cm non-structured scale
Acidity	Dislike extremely—Like extremely	15-cm non-structured scale
Aroma	Dislike extremely—Like extremely	15-cm non-structured scale
How do you feel when tasting this sample?	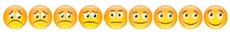	Face Scale (0–100)
Check all emojis that depict how you feel when tasting this sample	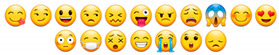	Check all that apply (CATA)
Check all emotions that depict how you feel when tasting this sample	Active/Joyful/Aggressive/Bored/Affectionate/Disgusted/Free/Friendly/Happy/Adventurous/Guilty/Nostalgic/Calm/Pleasant/Satisfied/Secure/Surprised/Worried *	Check all that apply (CATA)
Overall liking (rated at the end of the test)	Dislike extremely—Like extremely	15-cm non-structured scale

* Emotion-terms obtained from EsSense Profile^®^ [38].

**Table 3:** Physicochemical characterization of commercial beers.

Sample	Color						Density (g mL^−1^)	Viscosity (mPa s)	pH	Titratable Acidity
	L*	a*	b*	Hue	Chroma	YI				
LK	36.70 d† ± 0.12	23.68 b ± 0.08	17.76 c ± 0.05	0.64 b ± 0.001	29.60 c ± 0.09	69.15 c ± 0.39	1.02 a ± 0.001	2.16 a ± 0.11	3.17 d ± 0.01	0.41 a ± 0.01
LF	29.67 e ± 0.08	26.54 a ± 0.16	20.16 b ± 0.32	0.65 b ± 0.005	33.33 b ± 0.32	97.11 b ± 1.79	1.03 a ± 0.002	1.73 bc ± 0.06	2.94 e ± 0.01	0.32 b ± 0.03
C	59.36 a ± 0.07	−1.27 d ± 0.01	6.52 f ± 0.04	−1.38 c ± 0.003	6.64 f ± 0.03	15.69 f ± 0.07	1.00 c ± 0.003	1.48 d ± 0.09	4.29 b ± 0.00	0.11 d ± 0.00
H	58.72 b ± 0.25	−1.21 d ± 0.03	8.99 e ± 0.09	−1.44 d ± 0.002	9.07 e ± 0.09	21.87 e ± 0.21	1.00 c ± 0.002	1.80 b ± 0.07	4.31 b ± 0.01	0.10 d ± 0.01
L	56.68 c ± 0.27	−1.02 d ± 0.01	16.25 d ± 0.08	−1.51 e ± 0.001	16.28 d ± 0.08	40.96 d ± 0.09	1.01 b ± 0.002	1.54 cd ± 0.02	4.24 c ± 0.01	0.11 d ± 0.00
Z	26.58 f ± 0.16	16.82 c ± 0.14	37.28 a ± 0.52	1.14 a ± 0.003	40.90 a ± 0.53	200.40 a ± 1.59	1.00 c ±0.003	1.80 b ± 0.00	4.42 a ± 0.01	0.17 c ± 0.01

Abbreviations: CIELAB color parameters (L*: lightness, a*: red/green, b*: blue/yellow), YI: yellowness index. ^†^ Values represent the mean ± standard error (n_Titratable Acidity_ = 2, n_Color, Density, Viscosity, pH_ = 3). Abbreviations of samples may be found in Table 1. Different letters within a column indicate that values are significantly different according to the least significant difference test (LSD; *p* <0.05).

**Table 4:** Simple sugars, salt, total dissolved solids, ethanol content, and iso-α-acids of commercial beers.

Sample	Simple Sugars (mg mL^−1^)			Salt (%)	Total Dissolved Solids (ppm)	Alcohol Content (%)	Iso-α-Acids (mg L^−1^)	
	Glucose	Fructose	Maltose				Trans-Isocohumulone	Trans-Isohumulone
LK	13.91 a* ± 0.24	12.56 b ± 0.31	1.06 c ± 0.04	0.10 a ± 0.00	1148.00 b ± 11.00	3.53 e ± <0.001	0.33 e ± 0.01	0.45 d ± 0.01
LF	14.32 a ± 0.62	13.51 a ± 0.01	3.40 a ± 0.07	0.10 a ± 0.00	1226.00 a ± 7.00	2.53 f ± <0.001	0.22 e ± 0.01	0.38 d ± 0.01
C	ND	ND	ND	0.05 e ± 0.00	658.00 f ± 9.61	4.62 d ± <0.001	3.44 b ± 0.08	3.91 b ± 0.22
H	0.60 c ± 0.00	0.50 d ± 0.00	0.79 d ± 0.03	0.06 d ± 0.00	738.00 e ± 4.04	4.97 c ± <0.001	2.81 c ± 0.00	3.27 c ± 0.11
L	1.87 b ± 0.06	2.04 c ± 0.08	0.00 e ± 0.00	0.07 c ± 0.00	898.67 d ± 5.55	6.68 b ± <0.001	2.60 d ± 0.05	3.35 c ± 0.12
Z	ND	ND	2.97 b ± 0.12	0.09 b ± 0.00	1100.33 c ± 26.36	9.47 a ± <0.001	10.95 a ± 0.04	10.46 a ± 0.08

* Values represent the mean ± standard error (n_Simple sugars, Iso-α-acids_ = 2, n_Salt, Total dissolved solids, Ethanol content_ = 3). ND: Non-detectable. Abbreviations of samples may be found in Table 1. Different letters within a column indicate that values are significantly different according to the least significant difference test (LSD; *p* < 0.05).

**Table 5:** Sensory acceptability (self-reported responses) of commercial beers.

Sample	Overall Liking-Start	Foam Stability	Foam Height	Bitter	Sweet	Acidity	Aroma	Overall Liking-End
LK	10.35 a* ± 0.56	10.20 a ± 0.38	8.31 b ± 0.38	11.06 a ± 0.42	9.53 a ± 0.46	11.37 a ± 0.50	9.53 a ± 0.44	10.73 a ± 0.50
LF	10.04 ab ± 0.47	11.14 a ± 0.52	11.16 a ± 0.54	11.85 a ± 0.46	9.56 a ± 0.52	10.76 a ±0.51	9.50 a ± 0.50	10.79 a ± 0.50
C	7.57 cd ± 0.55	6.79 b ± 0.52	6.28 c ± 0.59	8.92 bc ± 0.45	8.62 a ± 0.53	7.07 b ±0.51	7.54 bc ± 0.53	7.74 bc ±0.54
H	8.72 bc ± 0.49	10.58 a ± 0.39	10.60 a ± 0.40	8.76 bc ± 0.51	9.51 a ± 0.51	7.35 b ±0.47	8.31 ab ± 0.51	9.07 b ± 0.49
L	6.69 d ± 0.58	10.63 a ± 0.43	10.32 a ± 0.41	7.78 c ± 0.57	6.61 b ± 0.58	6.91 b ±0.52	7.03 bc ± 0.55	6.90 c ± 0.63
Z	7.65 cd ± 0.63	10.46 a ± 0.51	10.83 a ± 0.44	9.48 b ± 0.60	6.83 b ± 0.61	7.59 b ±0.59	6.73 c ± 0.57	7.34 c ± 0.65

* Values represent the mean ± standard error *N* = 61. Different letters within a column indicate that values are significantly different according to the least significant difference test (LSD; *p* < 0.05). Abbreviations of samples may be found in Table 1.
